# Molecular mapping and characterization of the silkworm *apodal* mutant

**DOI:** 10.1038/srep18956

**Published:** 2016-01-07

**Authors:** Peng Chen, Xiao-Ling Tong, Ming-Yue Fu, Hai Hu, Jiang-Bo Song, Song-Zhen He, Ting-Ting Gai, Fang-Yin Dai, Cheng Lu

**Affiliations:** 1 State Key Laboratory of Silkworm Genome Biology, Southwest University, Chongqing 400716, China; 2 Key Laboratory for Sericulture Functional Genomics and Biotechnology of Agricultural Ministry, Southwest University, Chongqing 400716, China

## Abstract

The morphological diversity of insects is important for their survival; in essence, it results from the differential expression of genes during development of the insect body. The silkworm *apodal* (*ap*) mutant has degraded thoracic legs making crawling and eating difficult and the female is sterile, which is an ideal subject for studying the molecular mechanisms of morphogenesis. Here, we confirmed that the infertility of *ap* female moths is a result of the degradation of the bursa copulatrix. Positional cloning of *ap* locus and expression analyses reveal that the *Bombyx mori sister of odd and bowl* (*Bmsob*) gene is a strong candidate for the *ap* mutant. The expression of *Bmsob* is down-regulated, while the corresponding *Hox* genes are up-regulated in the *ap* mutant compared to the wild type. Analyses with the dual luciferase assay present a declined activity of the *Bmsob* promoter in the *ap* mutant. Furthermore, we demonstrate that *Bmsob* can inhibit *Hox* gene expression directly and by suppressing the expression of other genes, including the *BmDsp* gene. The results of this study are an important contribution to our understanding of the diversification of insect body plan.

The morphological diversity of insects makes them able to adapt rapidly to environmental changes and provides good models for studying body plan diversity^1^. The identification of key genes and the elucidation of pathways relating to the morphogenesis and pattern formation are of great interest. Insects share some common features; for example, the trunk is composed of segments that can be grouped into a head, a thorax and an abdomen. By contrast, insect appendages have morphological and functional differences despite the same basic types of appendage along the body axis^2^,^3^. Based on studies of *Drosophila*, genes involved in the formation of body segments can be divided into four conceptual categories; maternal effect, gap, pair-rule and segment polarity^4^,^5^,^6^. Segmental identity was determined along the anterior-posterior body axis by the homeotic (*Hox*) genes, whose expression patterns largely determine the morphology and development of tissues and organs^7^,^8^,^9^. Changes in *Hox* genes can lead to changes in insect body plan and morphology^1^,^10^. Despite our increased knowledge and understanding of insect morphogenesis, details of genes responsible for segmentation and their regulatory networks in non-*Drosophilia* insects remain obscure.

In *Drosophila*, the *odd-skipped* gene family consists of *odd-skipped* (*odd*), *brother of odd with entrails limited* (*bowl*), *sister of odd and bowl* (*sob*) and *drumstick* (*drm*), which have key roles during embryogenesis^11^,^12^,^13^. The *odd* gene, which was identified as a member of the pair-rule genes, encodes a C_2_H_2_ zinc finger transcription factor regulating the development of multiple tissues^14^,^15^,^16^,^17^,^18^. The *bowl* and *drm* genes are required for leg, notum and hindgut pattern formation^17^,^19^,^20^. The identification and analysis of related developmental mutants has contributed to our understanding of the *odd-skipped* family. Although recent studies suggested the *sob* gene might be involved in the development of antennae, legs and wings^21^,^22^,^23^,^24^, the particular role of this gene in insect morphogenesis is not clear.

Wings, ventral limbs and the reproductive system have pivotal roles in insects; hence, the identification of the corresponding developmental genes is of particular importance. In the silkworm *Bombyx mori*, the *apodal* (*ap*) mutant exhibits marked multiple morphological variations, including degraded thoracic legs and wings as well as female sterility, which is an ideal subject for the study of the molecular mechanisms for morphogenesis. The spontaneous *ap* mutant was identified by the presence of degraded thoracic legs throughout development and the *ap* adults exhibited smaller or deformed wings seriously affecting their ability to mate (Fig. 1). For all of these reasons, the *ap* mutant could not mate naturally and required artificial mating to produce normal offspring. Genetic analysis has revealed that it is located at 22.3 centimorgans (cM) on the third linkage in the silkworm genetic linkage map and controlled by a single recessive gene^25^. Moreover, the silkworm E complex mutants, located on chromosome 6 corresponding to the *Hox* genes, display ectopic legs, abnormal wings and genital system^25^,^26^, implying a relationship between the *ap* mutant and *Hox* genes.

In this study, we investigated the physiological cause of *ap* female infertility, and then identified and characterized the strong candidate gene responsible for the *ap* mutant. We confirmed that the infertility of *ap* female moths was due to the degradation of the bursa copulatrix, and demonstrated that the abnormal expression of the *Bmsob* gene encoding C_2_H_2_ zinc finger-containing transcriptional factor should be probably responsible for *ap* mutant. Finally, we found that the *Hox* genes in the *ap* mutant are abnormally expressed and verified the *Hox* genes can be regulated by the *Bmsob* gene.

## Results

### The female *ap* mutant exhibited degenerated bursa copulatrix

**Figure 1:**
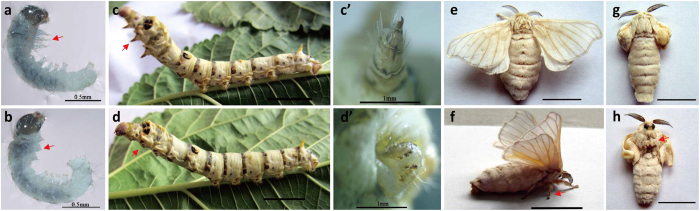
Phenotypes of wild type and *apodal* (*ap*) mutant. (**a**) Wild-type (*ap*/+^*ap*^) embryo. The arrow indicates the normal thoracic legs. Bar = 0.5 mm. (**b**) The *ap* mutant (*ap*/*ap*) embryo. The arrow indicates degraded thoracic legs. Bar = 0.5 mm. (**c**) Wild-type (*ap*/+^*ap*^) larva. The arrow indicates the normal thoracic legs. Bar = 1 cm. (**d**) The *ap* mutant (*ap*/*ap*) larva. The arrow indicates degraded thoracic legs. Bar = 1 cm. (**c′**) Enlargement of the thoracic leg in (**c**). Bar = 1 mm. (**d′**) Enlargement of the degraded thoracic leg in (**d**). Bar = 1 mm. (**e**) Dorsal side of the wild-type (*ap*/+^*ap*^) adult. Bar = 1 cm. (**f**) Lateral view of the wild-type (*ap*/+^*ap*^) adult. The arrow indicates the normal thoracic legs. Bar = 1 cm. (**g**) Dorasl side of the *ap* mutant (*ap*/*ap*) adult. Bar = 1 cm. The wings of the *ap* mutant adult were smaller compared to the wild type (**e**). (**h**) Ventral side of the *ap* mutant (*ap*/*ap*) adult. The arrow indicates degraded thoracic legs. Bar = 1 cm.

**Figure 2:**
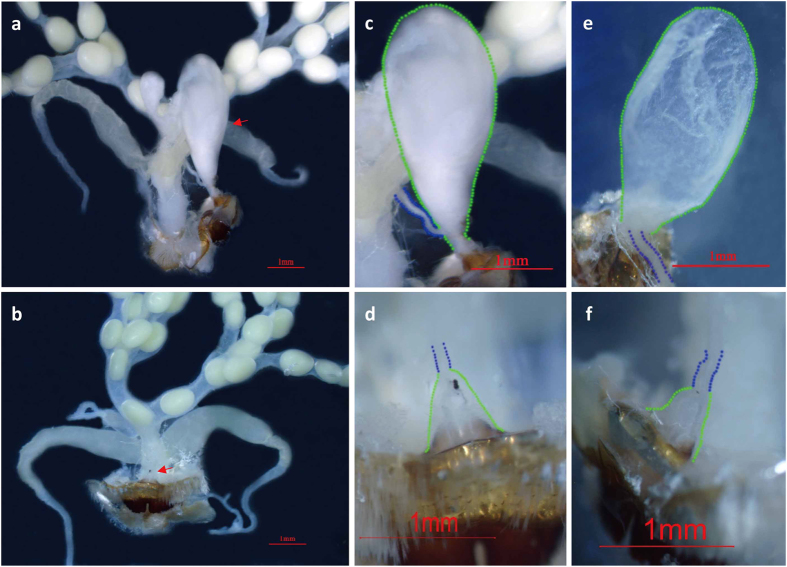
Genital glands of the wild type and *ap* mutant female adult. (**a**) The genital gland of the *ap*/+^*ap*^ female adult. The female moths were mated for 6 h before dissection. The arrow indicates the normal bursa copulatrix with seminal fluid. (**b**) The genital gland of the *ap*/*ap* female adult. The female moths were mated for 6 h before dissection. The arrow indicates the degraded bursa copulatrix. (**c**) Enlargement of the bursa copulatrix in (**a**). (**d**) Enlargement of the bursa copulatrix in (**b**). (**e**) Bursa copulatrix of the *ap*/+^*ap*^ female moth without mating. (**f**) Bursa copulatrix of the *ap*/*ap* female moth without mating. The green dots indicated the outline of the bursa copulatrix and the blue dots indicated the sperm canal. Bar = 1 mm.

In addition to the degraded thoracic legs and abnormal wings of the *ap* mutant (Fig. 1), another striking feature of the *ap* mutant was the female sterility, which had a significant effect on fertility. To explore the underlying cause of female sterility, further, we examined the reproductive system of *ap* mutant female moths from several aspects. We verified that the *ap* female moth could make eggs like the wild type, although the number of eggs varied more between *ap* individuals (Supplementary Fig. S1 and Table S1). We investigated the micropyle to make sure sperm can enter the egg smoothly and found that the micropyles of *ap* eggs were normal (Supplementary Fig. S2). We used artificial parthenogenesis on wild type and *ap* eggs and found about 50% of both can develop normally (Supplementary Fig. S3 and Table S2), suggesting that the contents of *ap* eggs are normal. According to these comparative experiments, our results indicated that *ap* female sterility was not caused by malformation of eggs. Next, we searched for evidence of structural variation in *ap* female internal genitalia. We found that the bursa copulatrix, which can store seminal fluid temporarily during the mating process, was degenerate in the *ap* mutant female moths (Fig. 2a,b). The normal bursa copulatrix of wild type *B. mori* is a sac-like structure and becomes club-shaped after mating (Fig. 2c,e), whereas the *ap* bursa copulatrix had only vestiges (Fig. 2d–f). Collectively, these studies revealed that *ap* female sterility was due to the degenerate structure of the bursa copulatrix.

### Positional cloning of the *ap* locus

**Figure 3:**
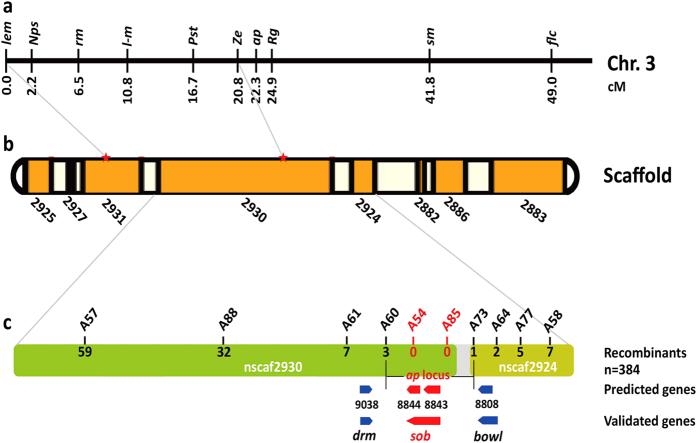
Mapping of the *ap* locus on the *B. mori* linkage group 3. (**a**) The 3^rd^ linkage group of *B. mori*. The mutation symbols are shown above the map and the corresponding locus in centiMorgans (cM) are shown below the map. (**b**) Genomic scaffolds on chromosome 6. Orange boxes represent the assembled scaffolds and the names of the scaffolds are shown below. (**c**) Scaffold map and gene model of the nscaf2930 and nscaf2924 scaffolds on chromosome 3. The markers are identified above the map and the numerals below the map indicate the number of recombinants identified in 384 F_2_ progeny. Blue arrows represent the predicted genes in SilkDB, and red arrows represent the validated genes.

We used a map-based cloning approach to investigate the *B. mori* genome sequence in an attempt to identify the gene responsible for the *ap* locus. In the *B. mori* linkage map, the *ap* locus was approximately 22.3 cM from the distal *lem* locus, which was reported to result from the mutation of *BmSpr*, a gene located on Scaffold 2931^25^,^27^ (Fig. 3a,b). In addition, the *Ze* locus was reported to be linked tightly with the FL0827 marker, which was located on Scaffold 2930^28^ (Fig. 3a,b). Hence, we used the *BmSpr* gene and FL0827 as anchor markers of the genome sequence to find new markers.

To map the *ap* locus more precisely, we generated an F_2_ population of 384 individuals with the *ap* mutant phenotype produced by selfing F_1_ progeny of a cross between a Dazao female and an *ap* male. We mapped the *ap* locus to a region between two markers, A60 and A73, and the markers A54 and A85 were linked tightly with the *ap* locus (Fig. 3c). Within the region between the markers A60 and A73, only two genes *BGIBMGA008843* and *BGIBMGA008844* were predicted to be present, while there is a gap region that has not been sequenced on the scaffolds (Fig. 3c). Both of the predicted genes had high levels of homology to *sister of odd and bowl* (*sob*) of *Drosophila melanogaster*, which is one member of the *odd-skipped* family that involved in *Drosophila* leg development^17^. Comparison and assembly of sequences of these two predicted genes and expressed sequence tags (ESTs) resulted in the dependable conclusion that they were actually one gene, designated as *Bombyx mori sister of odd and bowl* (*Bmsob*). Therefore, we next focused on the *Bmsob* gene that is the unique gene having sequence information within the mapped region.

Subsequently, according to the two predicted genes and ESTs, we obtained the full-length cDNA of *Bmsob* gene in the wild type and the *ap* mutant using rapid amplification of cDNA ends (RACE) technology, 1923 bp and 1924 bp, respectively (Supplementary Fig. S4). The length of the coding sequence region was 1311 bp, encoding 436 amino acids with five C_2_H_2_-type zinc finger structures. The 5′-untranslated region (5′-UTR) of both was 85 bp long, and the 3′-UTR was 527 bp and 528 bp long, respectively, and both had two exons. There were 15 single base mutations in *Bmsob* open reading frames (ORFs) between Dazao and the *ap* mutant, of which 13 were synonymous mutations and two were missense mutations (P → A, S → P). In addition, there were eight single base mutations and one base insertion in the *Bmsob* UTR in the *ap* mutant. In order to study the relationship between the candidate gene and the *ap* mutant further, we undertook a more detailed analysis of the *Bmsob* gene.

### Expression profiles of the *Bmsob* gene

**Figure 4:**
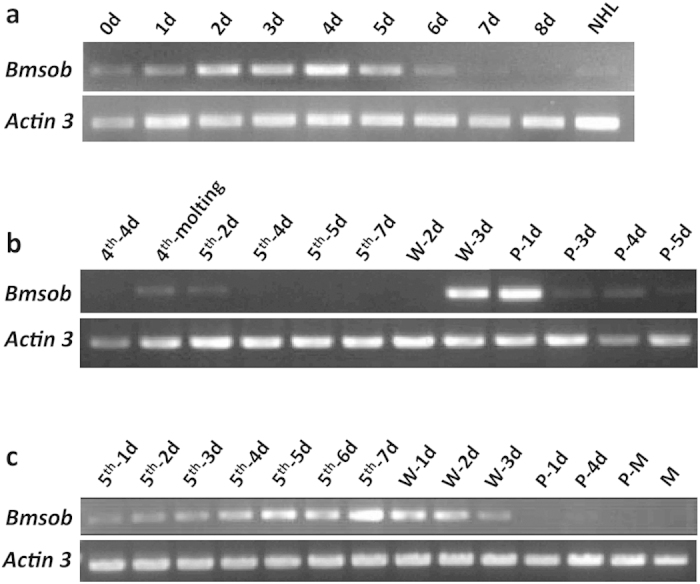
Temporal expression profiles of the *Bmsob* gene. (**a**) Expression profile of the *Bmsob* gene during silkworm embryonic development. NHL, newly hatched larvae. (**b**) Temporal expression of *Bmsob* in the silkworm from the 4^th^ instar larvae to the pupa stage. Total RNA samples were isolated from the whole bodies. (**c**) Temporal expression profile of the *Bmsob* gene in the development of the wing. Total RNA samples were isolated from the wing discs of larvae and the wings of pupae and moths. The *Bombyx mori Actin 3* gene was used as internal control. d, day; 4^th^, fourth instar; 5^th^, fifth instar; W, wandering stage; P, pupa stage; P-M, the day before the moth; M, moth.

Multiple defects associated with the *ap* mutant suggest its candidate gene is expressed in particular spatial temporal patterns. Therefore, we undertook a detailed analysis of the temporal expression pattern of the *Bmsob* gene. Given that the embryonic period is crucial for the development of limbs, we examined the temporal expression pattern of *Bmsob* mRNA at nine different developmental stages of the embryo and newly hatched larvae. The semi-quantitative RT-PCR analysis showed that expression of the *Bmsob* gene was detected first after oviposition and then increased gradually; it reached a peak level at 4 days after egg laying and decreased gradually thereafter (Fig. 4a). Remarkably, the high-level expression of *Bmsob* gene in stages 2, 3 and 4 days was consistent with silkworm limb development, suggesting that the *Bmsob* gene is involved in the early limb development.

As the silkworm undergoes complete metamorphosis and the late larval stage to pupal stage is especially important for metamorphosis, we examined the temporal expression pattern of *Bmsob* mRNA from the 4^th^ instar day 4 larva to the day 5 pupae. Our results showed that high-level expression of *Bmsob* gene appeared at the third day of the wandering stage and the first day of the pupal stage (Fig. 4b). This period is the key stage in the metamorphosis from larva to pupa and the important stage of genital gland development, implying that the *Bmsob* gene has an important role in the development of the silkworm genital gland. In addition, analysis of *Bmsob* gene expression pattern during wing development showed that the expression level of *Bmsob* increased gradually with development of the wing primordium (Fig. 4c). The level of *Bmsob* expression was highest in the final stage of the 5^th^ instar, implying that the *Bmsob* gene is involved in the development and formation of silkworm wings. Together, complex patterns of expression suggested extensive roles for the *Bmsob* gene during development of the silkworm.

### Expression level of *Bmsob* gene in the *ap* mutant compared to wild-type strain Dazao

**Figure 5:**
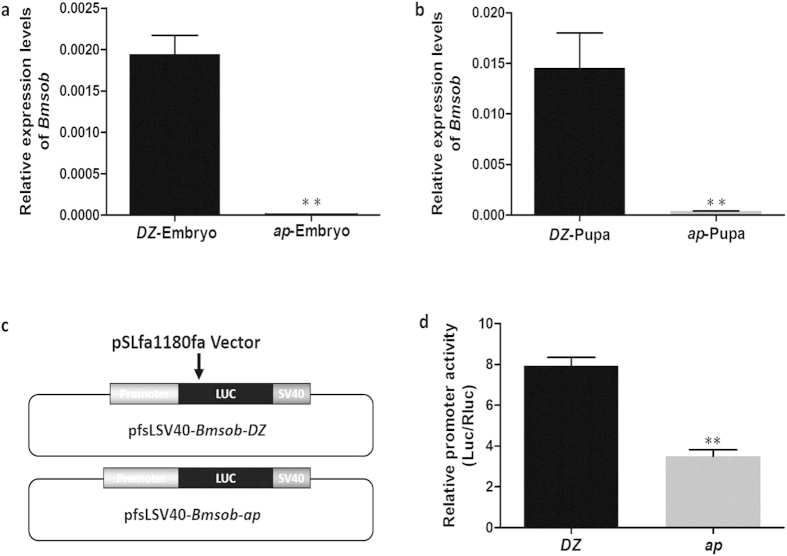
Analysis of the *Bmsob* gene and its promoter region in the wild type (Dazao) and the *ap* mutant. (**a**) Relative quantitative analysis of *Bmsob* in Dazao and *ap* mutant embryos. (**b**) Relative quantitative analysis of *Bmsob* in Dazao and *ap* mutant pupae (1 d). (**c**) A diagram of the construction of the luciferase reporter gene vectors. (**d**) The relative activity of the *Bmsob* promoter in Dazao and the *ap* mutant. *DZ*, Dazao; **P < 0.01, Student’s *t*-test, n = 3.

To assess the relationship between the *ap* mutant and the *Bmsob* mutated state, we compared the expression of *Bmsob* between *ap* and Dazao at two stages of development, day 4 of the embryo and the first day of pupation, when *Bmsob* normally showed its highest level of activity. The results of the quantitative RT-PCR (qRT-PCR) showed that the expression level of *Bmsob* was decreased markedly in the *ap* mutant compared to the wild type at both stages (Fig. 5a,b), while there was no significant difference in the expressions of *Bmdrm* and *Bmbowl* genes between the wild type and *ap* mutant (Supplementary Fig. S5). Because there was no significant difference in the coding region of the *Bmsob* gene in the *ap* mutant compared to the wild type, we examined the promoter region to explore the reason for the reduction of *Bmsob* expression in the *ap* mutant. We found sequence polymorphisms in the *Bmsob* promoter region in the *ap* mutant and in the Dazao stain (Supplementary Fig. S6). Then we detected the promoter activity in the wild type and the *ap* mutant using the dual luciferase report system, and found that the level of activity of the *Bmsob* promoter in the *ap* mutant was decreased significantly (Fig. 5c,d). Accordingly, we speculated that the sequence variation of the *Bmsob* promoter region might be a significant reason for the decreased expression level of the *Bmsob* gene.

### *Bmsob* is involved in maintenance of expression of the *Hox* genes

**Figure 6:**
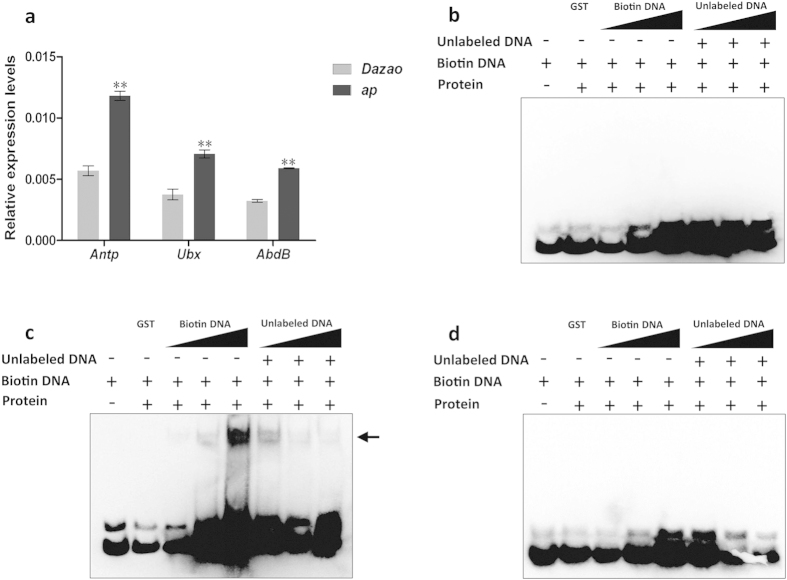
Expression profiles of *Hox* genes in Dazao and the *ap* mutant and the interaction between the Bmsob protein and *Hox* genes. (**a**) Expression profiles of *Hox* genes in Dazao and the *ap* mutant. **P < 0.01, Student’s *t*-test, n = 3. Detection by EMSA of (**b**) the interaction between the Bmsob protein and the *BmAntp* gene; (**c**) the interaction between the Bmsob protein and the *BmUbx* gene; (**d**) the interaction between the Bmsob protein and the *BmAbd-B* gene. The amount of GST protein used in the reactions was 1 μg and the amount of *Bmsob* protein was 0.8 μg. The amount of biotin labeling probe, in turn, was 1, 2 and 6 pmol. The amount of unlabeled probe, in turn, was 2, 20 and 100 pmol. The arrow indicates the protein-DNA complex.

The published results of studies on the functions of *Hox* genes led us to speculate that the appearance of abnormal thoracic legs, wings and bursa copulatrix in *ap* mutant might be related to the *B. mori Hox* genes. We used qRT-PCR to examine the expression levels of *Hox* genes *BmAntp*, *BmUbx* and *BmAbd-B*; the results showed that the expression of these three genes was up-regulated in the *ap* mutant compared to the wild type (Fig. 6a). To confirm that the *Bmsob* gene was responsible for the abnormal expression levels of these three *Hox* genes, we used electrophoretic mobility shift assay (EMSA) experiments to validate interactions between the Bmsob protein and the *BmAntp*, *BmUbx* and *BmAbd-B* genes. The results showed that the Bmsob protein could bind directly to the *BmUbx* gene but not to the *BmAntp* or *BmAbd-B* genes (Fig. 6b–d).

**Figure 7:**
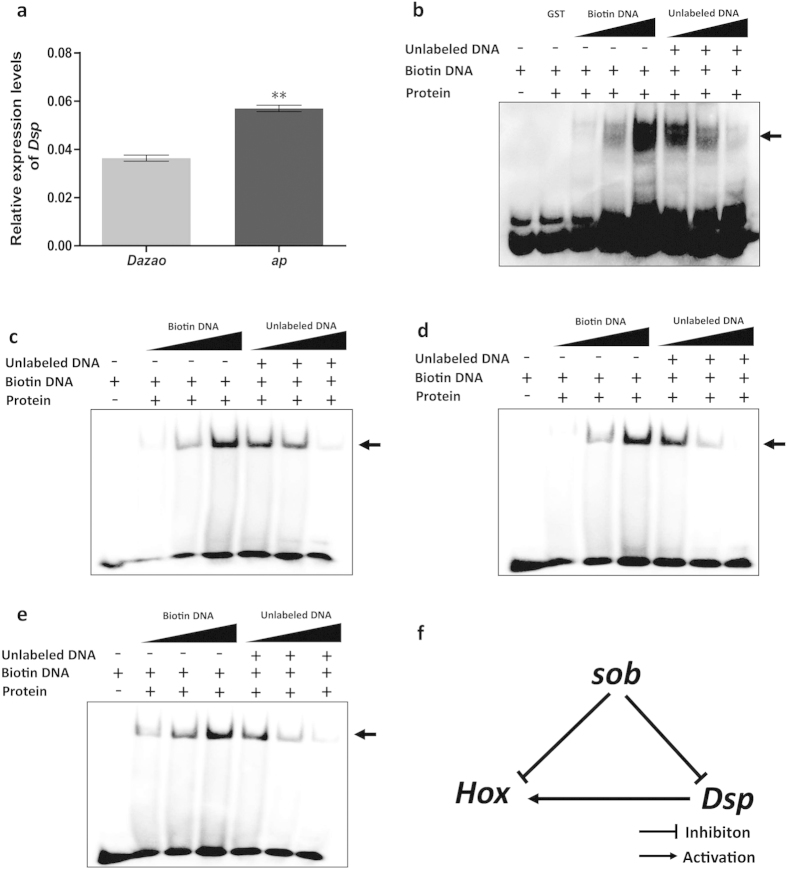
Expression profiles of the *BmDsp* gene in Dazao and the *ap* mutant, the interaction between the Bmsob protein and the *BmDsp* gene and the interaction between the BmDsp protein and the *Hox* genes. (**a**) Expression profiles of *BmDsp* genes in Dazao and the *ap* mutant. **P < 0.01, Student’s *t*-test, n = 3. Detection by EMSA of (**b**) the interaction between the Bmsob protein and the *BmDsp* gene; (**c**) the interaction between the BmDsp protein and the *BmAntp* gene; (**d**) the interaction between the BmDsp protein and the *BmUbx* gene; (**e**) the interaction between the *BmDsp* protein and the *BmAbd-B* gene. The amount of protein used in each reaction was: GST 1 μg; Bmsob 0.8 μg; *BmDsp* 0.8 μg. The amount of biotin labeling probe, in turn, was 1, 2 and 6 pmol. The amount of unlabeled probe, in turn, was 2, 20 and 100 pmol. The arrow indicates the protein-DNA complex. (**f**) Predicted regulation relationship between the *Bmsob*, *BmDsp* and *Hox* genes.

Further, in order to verify whether *Hox* genes can be regulated indirectly by the *Bmsob* gene, we screened the differentially expressed genes by microarray comparison of the wild type and the *ap* mutant (see Supplementary data for a complete list). Notably, the *Dsp* gene, which encodes a high-mobility group-like protein, appeared on our microarray list and exhibited a marked increase of *Dsp* expression in the *ap* mutant. The qRT-PCR result was consistent with the microarray data (Fig. 7a). In particular, its *Drosophila* homologous gene *dsp1* participated in the regulation of *Hox* genes^29^. The EMSA results confirmed that the Bmsob protein can bind to the *BmDsp* gene (Fig. 7b), and further results showed that the BmDsp protein can bind to the *BmAntp*, *BmUbx* and *BmAbd-B* genes (Fig. 7c–e). These findings led to the hypothesis that the *Bmsob* gene can inhibit *Hox* gene expression directly and indirectly through suppressing the expression of other genes, such as the *BmDsp* gene, which can promote expression of the *Hox* gene (Fig. 7f).

## Discussion

In this study, we demonstrated that the *Bmsob* gene is a strong candidate for the *B. mori ap* mutant which generates multiple defects by fine mapping and gene expression analysis. We confirmed that the infertility of *ap* female moths was due to the degradation of the bursa copulatrix. Moreover, we examined the relationship between the candidate gene *Bmsob* and the *Hox* genes, whose expression are essential for the proper organization of the animal body plan during development, indicating that *Bmsob* gene can inhibit *Hox* gene expression directly and indirectly through suppressing other genes, such as *BmDsp*. The results suggest that this regulatory network has an important role in the precise expression pattern of the *Hox* genes during development of the silkworm body plan.

The *odd-skipped* family genes encode zinc-finger transcription factors and are conserved in insects and vertebrates. Phylogenetic analysis showed that the silkworm-conserved *odd* gene family contained four genes belonging to four subfamilies, all of which had a single copy (Supplementary Fig. S7). Earlier studies showed that *odd* gene has widespread roles in embryonic development^16^,^17^,^18^, but few reports on the *sob* gene are available. Recent studies of *T. castaneum* by RNA interference (RNAi) screening showed that the *sob* gene might be involved in the development of legs and wings^22^,^24^. Similarly, microarray comparison analysis indicated that the *Drosophila sob* gene participates in the wing development^23^. Our results indicated that *Bmsob* as the only predicted gene on the scaffolds within our mapping region should be probably responsible for the multiple mutation phenotypes of the *ap* mutant, suggesting that *Bmsob* has important roles in the development of legs, wings and the reproductive system in the silkworm. Furthermore, the expression patterns of the *Bmsob* gene are consistent with the development of the embryo and wings (Fig. 4a,c). Especially, the massively high expression levels of the *Bmsob* gene occurred at the W-3d and P-1d stages, when the reproductive system was undergoing a violent metamorphosis (Fig. 4b). These data further implied an important role of the *Bmsob* gene during the development of these tissues and, according to its specific expression patterns in different tissues, we suggest that there are some gene-specific *cis*-regulatory elements for the *Bmsob* gene.

Although we have not identified large changes within the *Bmsob* gene in the *ap* mutant that predominantly affects its structure, we identified 15 single-base mutations in the *Bmsob* gene ORF between the wild type and the *ap* mutant, two of which cause missense mutations (P → A, S → P), and eight single base mutations and one base insertion into the UTR (Supplementary Fig. S4). Considering the decreased expression of the *Bmsob* gene in the *ap* mutant, we detected the promoter activity of *Bmsob* and the results showed that the activity of the *Bmsob* promoter in the *ap* mutant was decreased significantly (Fig. 5d). These data suggest that the *ap* mutant was not caused by large changes in the *Bmsob* coding region, such as deletions or insertions, but rather by changes with a few single nucleotide and/or in the *Bmsob* gene upstream regulatory sequences. Based on our mapping data, future studies will be aimed at identifying novel regulatory elements of the *Bmsob* gene in the responsible region. In addition, we found that the three genes, *drm*, *sob* and *odd*, were clustered in *D. melanogaster*, *Tribolium castaneum* and *Heliconius melpomene*^30^,^31^,^32^. Therefore, the *Bmodd* gene probably locates in the gap region. Considering of the fact that we did not obtain any significant phenotypes by *Bmsob* gene RNAi, we can’t rule out the possibility that the *Bmodd* gene may also contribute to *ap* mutant. On the basis of these results, our future research will pay some attention to test the possibility.

Numerous studies have demonstrated that the function of a *Hox* gene is related closely to its expression patterns^33^. In *Drosophila*, the *Antp*, *Ubx* and *Abd-B* genes were involved in development of the thoracic leg, wing and gonad respectively, which corresponds to their functional domain^34^,^35^,^36^. Similarly, our previous study suggested that the *Antp* gene is required for the organization of thoracic legs in *B. mori*^37^. These results suggested that multiple mutation phenotypes of the *ap* mutant might be related to the expression of the corresponding *Hox* gene, and provide clues for studying the mechanism underlying *Hox* gene expression. In the present study, our data showed that the expression of the *Antp*, *Ubx* and *Abd-B* genes was abnormal in the *ap* mutants, suggesting that there might be a regulatory relationship between them and the *Bmsob* gene. Subsequently, EMSA analysis revealed that the *Bmsob* gene could bind to the predicted transcription factor binding sites upstream of the *Ubx* gene (Fig. 6c), indicating that it might be regulated directly by the *Bmsob* gene. By contrast, our results showed that *Bmsob* might regulate the expression of *Hox* genes indirectly through other genes, such as *BmDsp*. Taken together, our results indicate that the product of the *Bmsob* gene would act as a repressor of target gene expression and the *Hox* genes, especially *Antp*, *Ubx* and *Abd-B* could be its important target genes in *B. mori*.

Uncovering the molecular genetic basis of mutations provides particularly significant clues for understanding the underlying mechanisms of animal development. Conspicuously, the recessive *ap* mutations have multiple developmental defects that are related to their survival and reproductive capacity, such as degraded thoracic legs, wings and bursa copulatrix, providing good targets for the study of pest control. In particular, studying female sterility can be used to make improvements to a highly effective area-wide method of pest control, known as the sterile insect technique^38^,^39^. Based on these results, our future work will concentrate on the inducement of pest sterility by genetic manipulation.

## Methods

### Silkworm strains and cell lines

The wild-type strain Dazao and the *ap* mutant strain (*ap*/*ap*) were obtained from the Silkworm Gene Bank at Southwest University, Chongqing, China. Silkworms were reared with fresh mulberry leaves at 25 °C under a photoperiod of 12h light/12h dark. The *B. mori* ovary cell line BmN-SWU1^40^ was cultured in TC-100 (US Biological) medium containing 10% (v/v) fetal bovine serum (Gibco), penicillin G (200 U/ml) and streptomycin sulfate (200 U/ml) at 27 °C.

### Scanning electron microscopy and artificial parthenogenesis

Eggs from non-mated females were dissected and rinsed with double-distilled water. For scanning electron microscopic observation, the eggs were processed as previously described^37^ and were coated with gold before observation under a scanning electron microscope (S-3000N, Hitachi, Tokyo, Japan). For artificial parthenogenesis, the newly dissected eggs were treated according to the following steps: 1) incubated at 25 °C for 12 h; 2) placed in the hot water at 46 °C for 18 min; 3) taken out of the water and incubated at 25 °C for 10 min; 4) transferred to 16 °C for 3 days; 5) treated with hydrochloric acid (sp. gr. 1.075) at 46 °C for 5 min; 6) incubated at 25 °C with adequate humidity.

### Positional cloning of the *ap* locus

Owing to the female sterility of the *ap* mutant, we used a single-pair cross between a Dazao female and an *ap* male to produce F_1_ offspring. Because there is no recombination in female silkworms, 22 progeny from a single-pair backcross between an F_1_ female and an *ap* male were used for the linkage analysis and 384 F_2_ progeny with the *ap* phenotype from selfing F_1_ (Dazao × *ap*) × (Dazao × *ap*) were used for the recombination analysis. Genomic DNA was extracted from parent moths, F_1_ moths and each F_2_ individual of third instar larvae as previously described^37^. Primer sets were designed from the *B. mori* genome sequence^41^ and the markers showing polymorphisms between Dazao and *ap* were used for linkage analysis (Supplementary Table S3) with MAPMAKER/EXP 3.0^42^ using the Kosambi function^43^. The bioinformatics analysis of candidate genes was done as previously described^37^.

### Rapid amplification of cDNA ends (RACE)

Total RNA of 7 days old Dazao embryos was isolated as previously described^37^. The 5′ and 3′ RACE was performed using Gene Racer Kit (Invitrogen, Carlsbad, CA, USA), according to the manufacturers’ protocol. PCR products were cloned into the pMD19-T vector (TaKaRa, Dalian, China) and sequenced. The primers used for PCR are listed in Supplementary Table S3.

### RT-PCR and quantitative RT-PCR (qRT-PCR)

To analyze gene expression patterns, the semi-quantitative RT-PCR experiments were performed. Total RNAs were isolated with TRIzol^®^ reagent (Invitrogen) from Dazao wild-type embryos of different developmental stages (from newly laid eggs to newly hatched larvae), the whole body of several developmental stages (from day 4 of the 4^th^ instar larvae to day 5 of the pupae) and the wing discs of larvae and the wings of pupae and moths (from day 1 of the 5^th^ instar larvae to the adult moth). The cDNA was synthesized using the PrimeScript reagent Kit with gDNA Eraser (TaKaRa) according to the manufacturer’s protocol. The gene for *Actin3* of *B. mori* was used as an internal control. For qRT-PCR, total RNA was isolated from 4 days old Dazao and *ap* embryos and reverse transcription was carried out as described above. The *ap*/*ap* embryos were from *ap*/+ females × *ap*/*ap* males and we distinguished *ap*/*ap* embryos from *ap*/+ by the apodal phenotype. The eukaryotic translation initiation factor 4A (silkworm microarray probe ID: sw22934) was used as an internal control. The qRT-PCR experiments were performed using the CFX96 Real-Time System (Bio-Rad, Hercules, CA, USA) with an iTaq Universal SYBR Green Supermix (Bio-Rad), according to the manufacturer’s recommended procedure. The specific primers for RT-PCR and qRT-PCR are listed in Supplementary Table S3.

### Phylogenetic analysis

Protein sequences were downloaded from the following websites: National Center for Biotechnology Information (NCBI) Protein Database (http://www.ncbi.nlm.nih.gov/protein), SilkDB^41^ (http://silkworm.genomics.org.cn/), Flybase^30^ (http://flybase.org/), BeetleBase^31^ (http://beetlebase.org/), VectorBase^44^ (https://www.vectorbase.org/) and *Heliconius* Genome^32^ (http://www.butterflygenome.org/). Accession numbers are listed in Supplementary Table S4. A multiple sequence alignment of the complete protein sequences was performed using MUSCEL program with default parameters. The phylogenetic tree was constructed using the Bayesian approach. Bayesian inferences were performed using MrBayes v3.1.2^45^ with the VT model estimated by protest-3.2 software^46^. We performed 2,500,000 generations and other parameters were set as default. The phylogenetic tree was visualized with MEGA 6^47^.

### Dual luciferase reporter assays

We constructed recombinant plasmids from a pfsLSV40 vector (a gift from Dr. Wang, Southwest University) that contained the *luciferase* gene driven by a *Bmactin4* promoter. The promoter region of the *sob* gene amplified from the Dazao and *ap* strains was cloned into pfsLSV40 to replace the *Bmactin4* promoter, respectively. Primers are listed in Supplementary Table S3. The resulting pfsLSV40-Bmsob-Dazao and pfsLSV40-*Bmsob-ap* plasmids were used for analyzing the promoter activity. BmN-SWU1 cells (1 × 10^6^) were seeded in 24-well culture plates (Corning Glass Works, Corning, NY, USA). After 24 h, cells were co-transfected with 400 ng pfsLSV40-*Bmsob*-Dazao plasmid or pfsLSV40-*Bmsob-ap* plasmid and 100 ng pIZ-*Rluc* control plasmid. At 48 h after transfection, a GloMax-Multi Detection System and a Dual-Glo Luciferase Assay Kit (Promega, Madison, WI, USA) were used to quantify luciferase activity. All assays were performed in triplicate.

### Microarray analysis

Total RNA was extracted from the wild type and *ap* mutant embryos as described above. For the hybridization experiment, we mixed equal amounts of total RNA from at least seven biological replicates to create one sample. Microarray hybridization and raw data normalization were carried out by Capital Bio Corp. (Beijing, China) as described^48^. For the microarray data analysis, a gene was considered as up- or downregulated in the *ap* mutant compared to the wild type if it displayed at least a twofold change.

### Recombinant expression and protein purification

The ORF of *Bmsob* and *BmDsp* were cloned into the pGEX-4T-1 vector with a glutathione *S*-transferase (GST) tag and pET-28a (+) vector with a His tag, respectively. Primers are listed in Supplementary Table S3. The proven clones were transformed into *Escherichia coli* strain BL21 to express GST-tagged Bmsob (GST-sob) and His-tagged BmDsp (His-Dsp) proteins. The GST-sob protein and His-Dsp protein were purified using a GSTrap FF column and a HisTrap HP column (GE Healthcare, Freiburg, Germany), respectively, according to the manufacturers’ protocol. Purified proteins were verified through western blotting as described previously^49^ with anti-GST tag and anti-His tag antibodies (Beyotime, Jiangsu, China), respectively.

### Electrophoretic mobility shift assay (EMSA)

For the prediction of *Bmsob* and *BmDsp* binding sites, we used two different transcription factor binding site prediction programs, Tfsitescan (http://www.ifti.org/cgi-bin/ifti/Tfsitescan.pl) and PROMO (http://alggen.lsi.upc.es/cgi-bin/promo_v3/promo/promoinit.cgi?dirDB=TF_8.3). The features related to the gene binding sites are G+C-rich and A+T-rich sequences for *Bmsob* and *BmDsp*, respectively, in accordance with their structural domains^50^,^51^. For EMSA, the probes were 5′-labeled by using biotin (Invitrogen) and then the labeled oligonucleotides were annealed to produce a double-stranded probe. The probes are listed in the Supplementary Table S3. The EMSA assay was performed with a LightShift Chemiluminescent EMSA Kit (Pierce, Rockford, IL, USA) according to the manufacturers’ protocol. After incubation, reaction products were loaded on to 5% (w/v) polyacrylamide non-denaturing gel (acrylamide:bis-acrylamide 19/1, w/w) and electrophoresed in TBE buffer (89 mM Tris, 89 mM boric acid, 2 mM EDTA, pH 8.3) for approximately 1.5 h at 100V. Then, the protein-DNA complexes were transferred electrophoretically from the gel onto nylon membranes (Invitrogen) for approximately 45 min at 380 mA, and visualized with streptavidin-horseradish peroxidase conjugate (Pierce) according to the manufacturer’s protocol.

## Additional Information

**How to cite this article**: Chen, P. *et al.* Molecular mapping and characterization of the silkworm *apodal* mutant. *Sci. Rep.* **6**, 18956; doi: 10.1038/srep18956 (2016).

## Supplementary Material

Supplementary Information

Supplementary Dataset 1
