# Supplementary material for: Molecular mapping and characterization of the silkworm apodal mutant
Source: Sci Rep. 2016 Jan 7;6:18956. doi: 10.1038/srep18956 (PMC4704060; doi:10.1038/srep18956)
Supplement: Supplementary Information [file srep18956-s1.pdf]

Molecular mapping and characterization of the silkworm *apodal* mutant

Peng Chen[1, 2+], Xiao-Ling Tong[1+], Ming-Yue Fu[1], Hai Hu[1], Jiang-Bo Song[1], Song-Zhen He[1], Ting-Ting Gai[1], Fang-Yin Dai[1, 2*], Cheng Lu[1, 2*]

[1]State Key Laboratory of Silkworm Genome Biology, Southwest University, Chongqing 400716, China

[2]Key Laboratory for Sericulture Functional Genomics and Biotechnology of Agricultural Ministry, Southwest University, Chongqing 400716, China

[*]Corresponding authors:

Cheng Lu: State Key Laboratory of Silkworm Genome Biology, Southwest University, No.216 Tiansheng Road, BeiBei District, Chongqing, 400716, China. Tel.: 86-23-68250346; Fax: 86-23-68251128; E-mail: lucheng@swu.edu.cn.

Fang-Yin Dai: State Key Laboratory of Silkworm Genome Biology, College of Biotechnology, Southwest University, No.216 Tiansheng Road, BeiBei District, Chongqing, 400716, China. Tel.: 86-23-68250793; Fax: 86-23-68251128; E-mail: fydai@swu.edu.cn.

[+]These authors contributed equally to this work.

Supplementary Figures

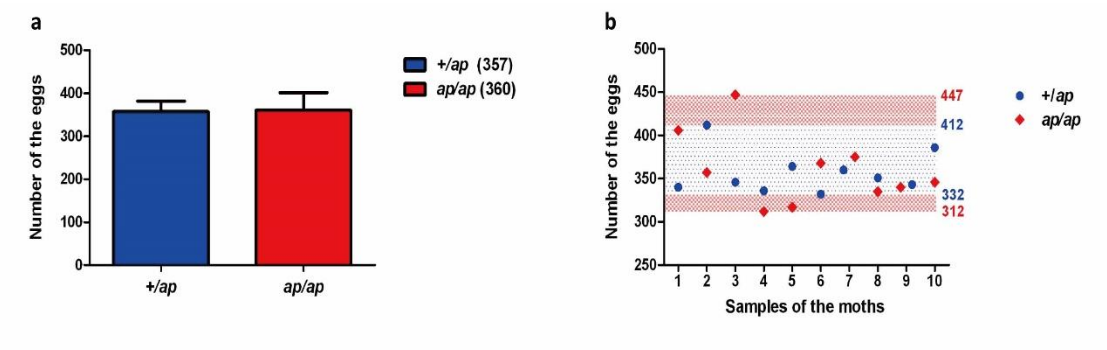

Figure S1. Investigational result of eggs from wild type and *ap* mutant female moths. (a) Comparison of average eggs from wild type and *ap* mutant female moths. (b) Distribution of individual eggs from wild type and *ap* mutant female moths.

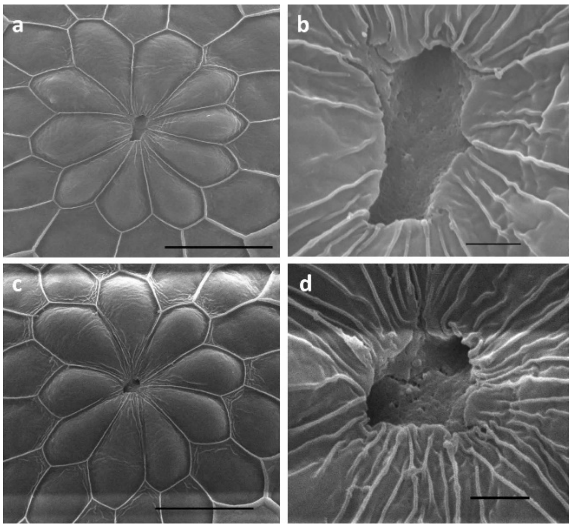

Figure S2. Micropyle of wild type and *ap* mutant eggs. (a) Micropyle of +/*ap*. Bar = 20 μm. (b) Enlargement of the micropyle in (a). Bar = 2 μm. (c) Micropyle of *ap*/*ap*. Bar = 20 μm. (d) Enlargement of the micropyle in (c). Bar = 2 μm.

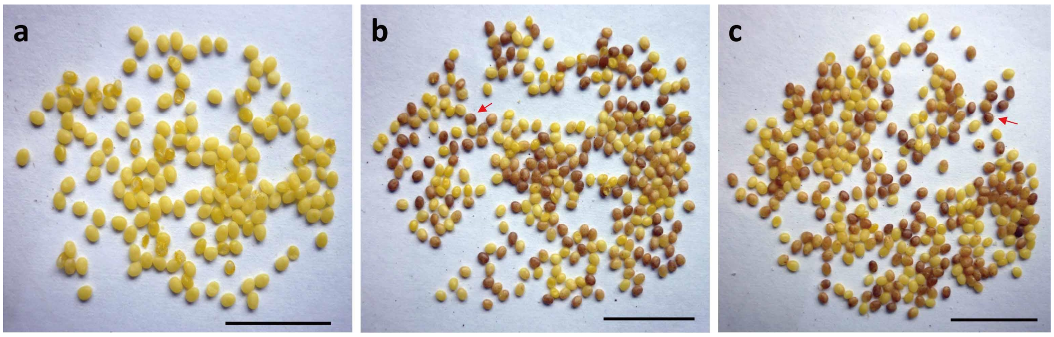

Figure S3. Artificial parthenogenesis of eggs from the wild type and *ap* mutant. (a) Untreated eggs. (b) Treated eggs from +/*ap*. (c) Treated eggs from *ap*/*ap*. The arrows indicate the eggs changing color. Bar = 1cm.

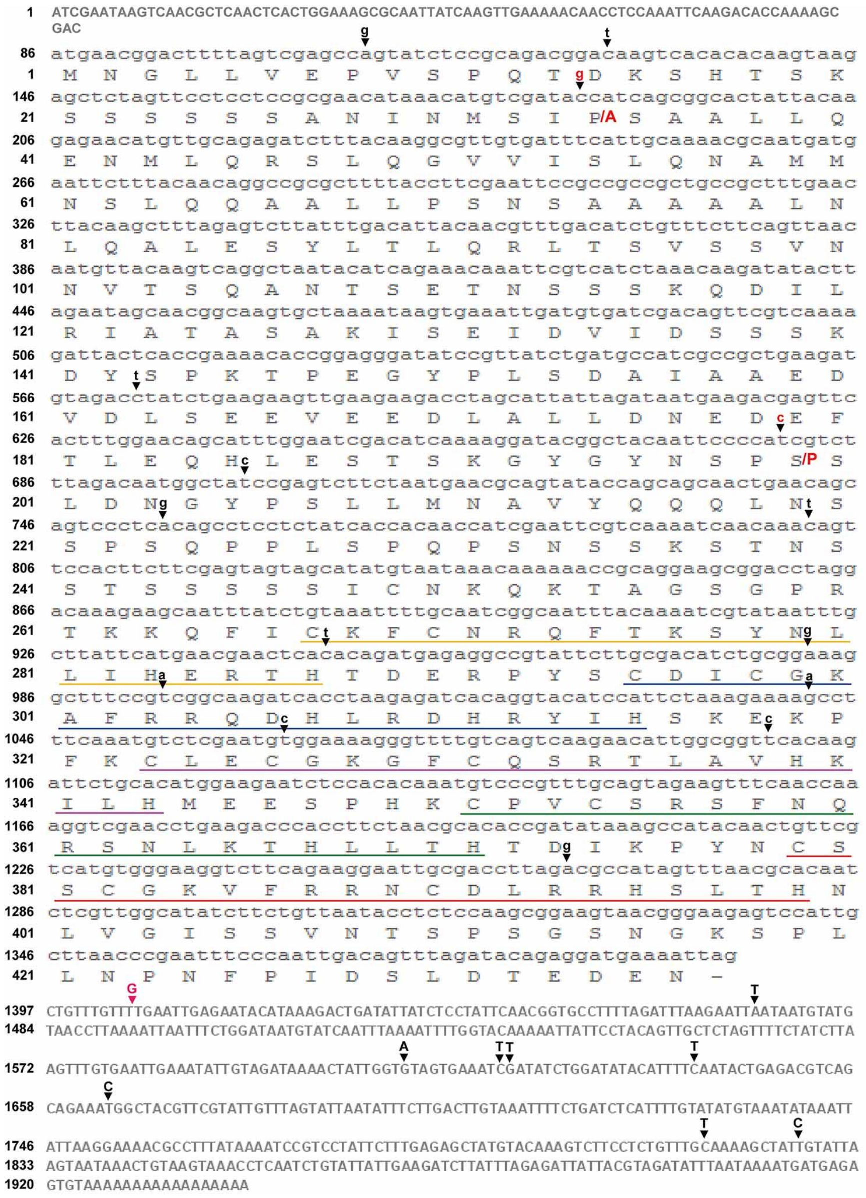

Figure S4. Nucleotide and deduced amino acid sequences of the *Bmsob* gene in the wild type (Dazao) and the *ap* mutant. The cloned nucleotide sequence is shown above the deduced amino acid sequence. Black arrows indicate single nucleotide mutations in the *ap* mutant. The pink arrow indicates the single nucleotide insertion. The mutated nucleotides are shown above the arrows. The amino acids colored red represent the amino acid mutations resulting from the mutation of a single nucleotide in the *ap* mutant. The colored lines indicate the sequences of five C_2H_2 zinc finger structures.

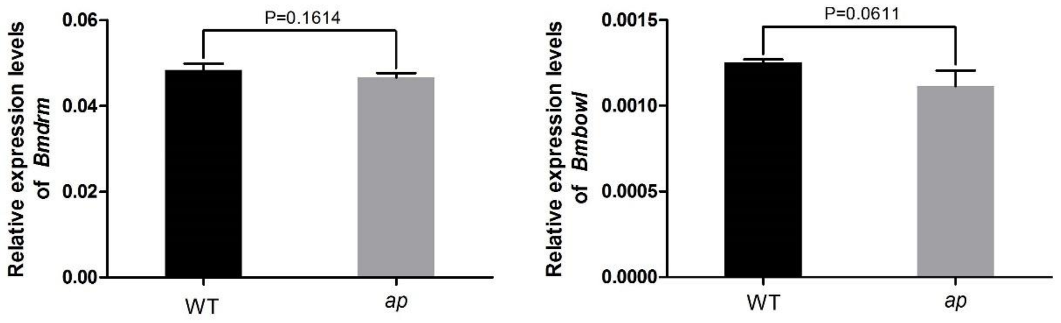

Figure S5. Relative quantitative analysis of *Bmdrm* and *Bmbowl* genes in the wild type and *ap* mutant embryos. Student's *t*-test, n = 3.

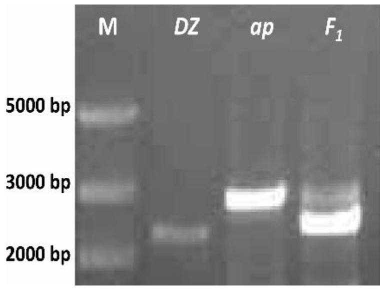

Figure S6. Polymorphism of the *Bmsob* promoter region in Dazao and the *ap* mutant. M, DNA marker; *DZ*, Dazao.

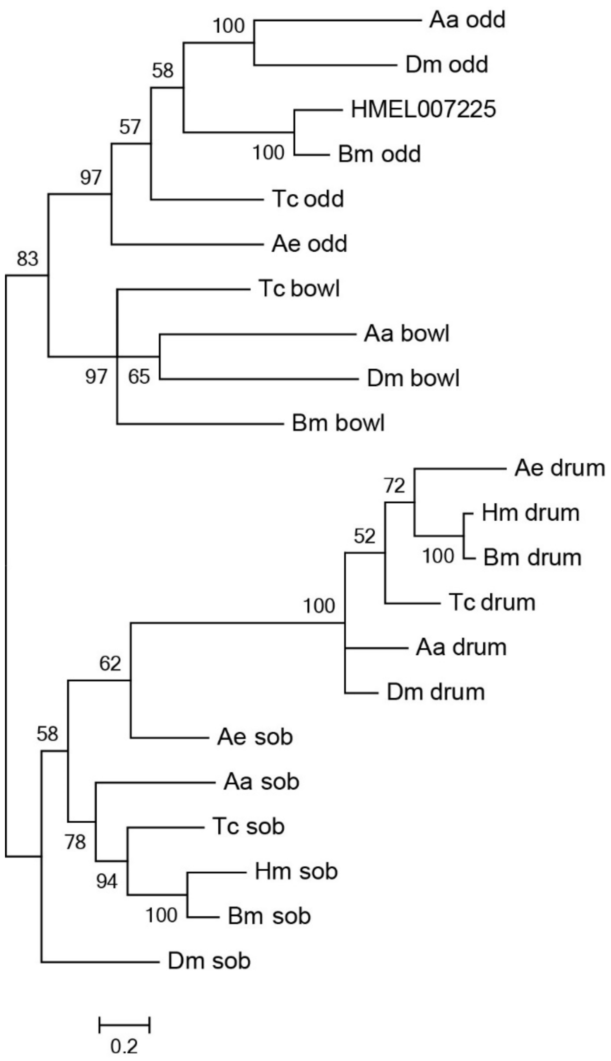

Figure S7. Phylogenetic tree based on complete protein sequences of the *odd-skipped* family in *B. mori* and other species. The phylogenetic tree was constructed using the Bayesian approach and was visualized with MEGA 6 software. Aa, *Aedes aegypti*; Ae, *Acromyrmex echinatior*; Bm, *Bombyx mori*; Dm, *Drosophila melanogaster*; Hm, *Heliconius melpomene*; Tc, *Tribolium castaneum*.

Supplementary Tables

Table S1. The number of eggs produced by normal and *ap* mutant female moths

Strain	1	2	3	4	5	6	7	8	9	10	Mean	SD	R
+/*ap*	340	**412**	346	336	364	**332**	360	351	343	386	357	25.02	80
ap/*ap*	406	357	**447**	**312**	317	368	375	335	340	346	360	41.31	135

Table S2. The results of artificial parthenogenesis

Strain	Untreated			Treated		
	No. of experimental eggs	No. of color-turning eggs	Percentage of color-turning eggs	No. of experimental eggs	No. of color-turning eggs	Percentage of color-turning eggs
+/*ap*	200	0	0	338	168	49.70%
				342	183	53.51%
				369	192	52.03%
ap/*ap*	200	0	0	326	151	46.32%
				357	185	51.83%
				331	174	52.57%

Table S3. The primer sets used in this study

Object	*Primer name*	*Sense sequence (5′-3′)*	*Antisense sequence (5′-3′)*
Linkage analysis	A54	GCAATGGACTCTTCCCGTTACT	GGAGAGTAAGCCACGGAGTTCA
	A57	GATGTCCCTTCTCACGCTTTG	ATATCACCTACCGTCCAACGAC
	A58	TTTGAACCTTATTGTTGTGCCT	AAAAAGTGACGAAGAAGGAGAAT
	A60	GACCTGGGGAGGACTTGTGAA	CGGGTTCAGCGAAAGATTGC
	A61	AAAGCCGCTTGCTCTGTAGT	AAAGGAAACTGGGATAGGAATG
	A64	CCCTCTTCAGGACGAACCAGC	GCATCGCCCGATACAACCA
	A73	TGGGTTCAGGATTTTGATGTAAG	GTAGTGACCAAGTGTCCGAGCA
	A77	GATTCTCGGGCGGAGTCAGTT	CGAAAGCCACTCCATCCCTG
	A85	TAGCAGGTCCCACAAATCAC	TTGGGTTTCAATGTTGTTCTG
	A88	CTTTTCGTATCTGTCAGCAACT	AGTCATTTCGATTCTGGCAG
RACE	*Bmsob* 5′ Primer	CGACTGGAGCACGAGGACACTGA	GCTTCCTGCGGTTTTTTGTTTATTAC
	Bmsob 5′ Nested Primer	GGACACTGACATGGACTGAAGGAGTA	CTGCGTTCATTAGAAGACTCGGATAG
	Bmsob 3′ Primer	TTTTGTCAGTCAAGAACATTGGCGGT	GCTGTCAACGATACGCTACGTAACG
	Bmsob 3′ Nested Primer	GGAAGTAACGGGAAGAGTCCATTGC	CGCTACGTAACGGCATGACAGTG
RT-PCR	RT-*Bmsob*	AACGGCAAGTGCTAAAATAAGT	GTTCTTGACTGACAAAACCCTT
	Actin3	AACACCCCGTCCTGCTCACTG	GGGCGAGACGTGTGATTTCCT
qRT-PCR	qRT-*Bmsob*	TTCTTGCGACATCTGCGGA	CGACGAACAGTTGTATGGCTTTA
	BmDsp	GAGGTGGAATACAATGTCGGAA	TGGCATTAGGGTCTTTCATCTG
	BmAntp	GAGACACCGTCCGTCAACAAC	CGTATTTCCGTCGCAGATTTT
	BmUbx	CCACCAGCCTCGTCCTCA	CTTTCCCACCATAGCCATTCT
	BmAbd-B	TTCTCTGTCAATGTCGGTCGG	GCTCTTCACTTGTCTCTCGGTTAG
	Bmdrm	ACACGGGATTTCAGACGCAA	AGAGGTTGTCTTGCCGCTTG
	Bmbowl	TGTCTGATGGGTCTCCGCAA	GCCGCTCGTCTGTATGGGTT
	sw22934	TTCGTACTGCTCTTCTCGT	CAAAGTTGATAGCAATTCCCT
Promoter activity analysis	Promoter	ACGCGTCGACGTTTGAACAATGACGTCATTTT	CGCGGATCCGTCGCTTTTGGTGTCTTGAA
Recombinant expression	Bmsob	CGCGGATCCATGAACGGACTTTTAGTCGAG	CCGCTCGAGTTAATTTTCATCCTCTGTATCTAAAC
	BmDsp	CGCGGATCCATGCTACAGCAGCATCAACAGC	CCGCTCGAGTCAATTGTATTCTTCGTCTGCATC
EMSA	Bmsob-*BmAntp*	GCCCTTGGGGCCGAAAATCTG	CAGATTTTCGGCCCCAAGGGC
	Bmsob-*BmUbx*	CGCGCTCCCGCCACACGCGAC	GTCGCGTGTGGCGGGAGCGCG
	Bmsob-*BmAbd-B*	GCCTCGGCGCGCCCTGCGACG	CGTCGCAGGGCGCGCCGAGGC
	Bmsob-*BmDsp*	AATATCCCTCGCCTAGTTACGCTGGC	GCCAGCGTAACTAGGCGAGGGATATT
	BmDsp-*BmAntp*	GCGACGGAAAATACGGACGT	ACGTCCGTATTTTCCGTCGC
	BmDsp-*BmUbx*	GATAAGGAAAGGAAAAGAGAG	CTCTCTTTTCCTTTCCTTATC
	BmDsp-*BmAbd-B*	GAAAAATTGTTTTACCTGGAACTATA	TATAGTTCCAGGTAAAACAATTTTTC

Table S4. Accession numbers of the genes presented in this study

Gene Symbol	Database	Accession Number
Bm_drm	GenBank	KT254333
Bm_sob	GenBank	KT254334
Bm_bowl	GenBank	KT254331
Bm_odd	GenBank	KT254332
Bm_Dsp	GenBank	KT254335
Dm_drm	GenBank	AHN54105
Dm_sob	GenBank	AAC47282
Dm_odd	GenBank	AAF51085
Dm_bowl	GenBank	AFH03539
Ae_drm	GenBank	XP_011054714
Ae_odd	GenBank	XP_011054717
Ae_sob	GenBank	EGI65373
Tc_drm	GenBank	XP_008196752
Tc_odd	GenBank	EFA09193
Tc_bowl	GenBank	EFA09192
Tc_sob	GenBank	EFA09194
Hm_drm	*Heliconius* Genome	HMEL007217
Hm_sob	*Heliconius* Genome	HMEL007222
HMEL007225	*Heliconius* Genome	HMEL007225
Aa_drm	VectorBase	AAEL004283
Aa_odd	VectorBase	AAEL007450
Aa_bowl	VectorBase	AAEL008961
Aa_sob	VectorBase	AAEL007453
